# Randomized Efficacy and Safety Trial with Oral S 44819 after Recent ischemic cerebral Event (RESTORE BRAIN study): a placebo controlled phase II study

**DOI:** 10.1186/s13063-020-4072-2

**Published:** 2020-02-03

**Authors:** Hugues Chabriat, Claudio L. Bassetti, Ute Marx, Françoise Picarel-Blanchot, Aurore Sors, Celine Gruget, Barbara Saba, Marine Wattez, Marie-Laure Audoli, Dirk M. Hermann

**Affiliations:** 1 Department Neurology, Lariboisière Hospital, APHP and University Denis Didierot, Paris 7, Paris, France; 2 FHU Neuro Vasc, Paris, France; 3 INSERM U1141, Paris, France; 4 Inselspital Bern, Bern, Switzerland; 5 Institut de Recherches Internationales Servier (IRIS), 50 rue Carnot, 92284 Suresnes Cedex, France; 6 Chair of Vascular Neurology, Dementia and Ageing Research, Department of Neurology, University Hospital Essen, Hufelandstraße 55, 45122 Essen, Germany

**Keywords:** S44819, Ischemic cerebral stroke, Efficacy, Safety, Study design, GABA

## Abstract

**Background:**

The GABA_A_-α5 receptor antagonist S44819 is a promising candidate to enhance functional recovery after acute ischemic stroke (IS). S44819 is currently evaluated in this indication; RESTORE brain study started in Dec 2016 and was completed in March 2019.

**Methods/design:**

The study is a 3-month international, randomized, double-blind, parallel group, placebo-controlled phase II multicentre study. Patients in 14 countries who suffered an IS leading to a moderate or severe deficit defined by NIHSS score ranging from 7 to 20 and are aged between 18 to 85 years are included between 3 and 8 days after the stroke onset. Approximately 580 patients are to be included.

The primary objective of the study is to demonstrate the superiority of at least one of the two doses of S44819 (150 or 300 mg bid) compared to placebo on top of usual care on functional recovery measured with the modified Rankin scale at 3 months. Comparisons between two doses of S44819 and placebo are assessed with ordinal logistic regression evaluating the odds of shifting from one category to the next in the direction of a better outcome at day 90. Secondary objectives include the evaluation of S44819 effects on neurological examination using the National Institute of Health Stroke Scale total score, activities of daily living using the Barthel Index total score, and cognitive performance using the Montreal Cognitive Assessment scale total score and Trail Making Test times. Safety and tolerability of the two doses of S44819 will also be analyzed.

**Discussion:**

The RESTORE BRAIN study might represent the first proof of concept study of an innovative therapeutic approach that is primarily based on enhancing functional recovery after IS.

**Trial registration:**

Randomized Efficacy and Safety Trial with Oral S 44819 after Recent ischemic cerebral Event, an international, multi-centre, randomized, double-blind placebo-controlled phase II study. ClinicalTrials.gov, NCT02877615; Eudract 2016–001005-16. Registered 24 August 2016

## Background

Post-stroke recovery relies on brain neuroplasticity [1, 2], which is compromised by the sustained hypoexcitability observed in the peri-infarct cortex due to the increased activity of GABAergic neurons [3].

S44819 is a potent and selective antagonist of GABA_A_-α5 receptors [4] that enhances motor and cognitive recovery when administered chronically from day 3 after IS in rodent models [5]. A transcranial magnetic stimulation study has demonstrated in humans that S44819 reaches the human cortex and is capable of increasing cortical and cortico-spinal excitability by reducing GABA_A_ receptor-mediated activity [6].

The design of a phase II clinical trial testing the efficacy of two doses of S44819 on functional recovery after acute supratentorial IS is presented.

## Methods/design

### Design

**Fig. 1:**
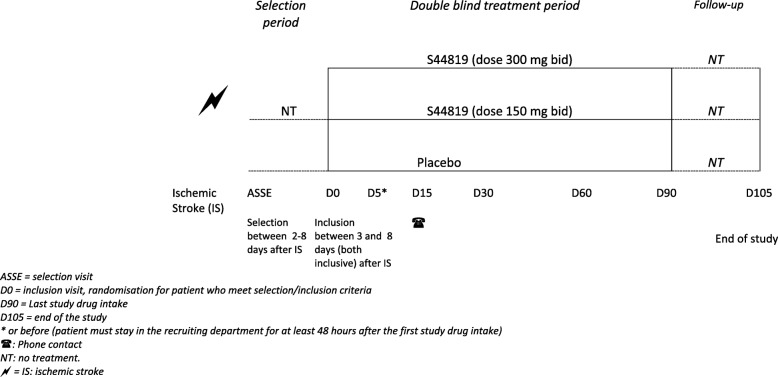
Treatment arms

In accordance with the EMA’s guidance on stroke [7], the study includes a selection period and a double-blind treatment period of 90 days. A follow-up period of 15 days after the end of treatment allows evaluation of the safety of patients (Fig. 1).

### Patient population

Male or female patients aged 18–85 years are randomised between 3 and 8 days after the stroke event based on the following criteria: 1) acute IS confirmed by MRI or CT; 2) NIHSS score between 7 and 20 both inclusive [8, 9]; 3) lack of previous disability defined as an estimated prestroke mRS score 0–1; 4) written informed consent.

Patients are ineligible if: 1) an acute hemorrhagic stroke or symptomatic hemorrhagic transformation or cerebral venous thrombosis occurs; 2) the required rehabilitation is impossible to undertake; 3) a carotid endarterectomy is planned within the next 3 months; 4) a previous clinically significant condition interferes with the study evaluation; 5) brain imaging shows a severe microangiopathy (Fazekas grade 3 with severe small vessels disease on MRI or CT scan); 6) receiving a treatment that could have an impact on treatment efficacy (i.e., interacting with GABA-A receptors) or a clinically relevant abnormality likely to interfere with study outcome, such as any renal or hepatic severe impairment and any repeated QTcF prolongation. Patients who are not able to cooperate as well as those presenting with geographic or social factors making their study participation impossible are excluded.

### Setting

The study is conducted in 92 hospital stroke centers in 14 countries (Australia, Belgium, Brazil, Canada, Czech Republic, France, Germany, Hungary, Italy, Poland, South Korea, Spain, The Netherlands, United Kingdom) that have experienced specialists in stroke medicine (e.g., are able to perform intravenous thrombolytic therapy, brain imaging for diagnosis). At discharge from hospital stroke centers, patients receive rehabilitation therapy (in- or out-patient rehabilitation) in accordance with the standard of care in each country and their clinical status. Patients are followed by the enrolling investigators.

### Randomization

The treatment is assigned at the inclusion visit by a balanced, non-adaptive randomization, with stratification by country and previous revascularization therapy status (thrombolysis and/or endovascular therapy). Patients are randomized into one of the three groups: S 44819 (150 mg or 300 mg twice a day) or placebo to reach about 194 patients per treatment arm. Randomization and allocations are centralized by Interactive Web Response System (IWRS) under blind conditions for subjects, caregivers, investigators, study-related staff, and sponsor. The placebo is made up of hydroxypropyl methylcellulose acetate succinate and different iron oxide colorants and is an off-white to yellow powder in a sealed sachet for oral suspension. Investigational Medicinal Product (IMP) is provided in the form of sachets (two sachets per intake) with an identical appearance and taste for all treatment groups. The circumstances under which blinding may be broken in IWRS are any serious AEs or severe medical conditions where the knowledge of the treatment is necessary for safety follow-up of the patient**.**

The Methodology and Analysis of Clinical Data Division of I.R.I.S is responsible for designing and constructing the blinded randomization list.

### Treatment

Study treatments are S 44819 (150 or 300 mg/bid) and placebo. The choice of doses is mainly based on a Transcranial Magnetic Stimulation study which has demonstrated that S 44819—at least at doses > 100 mg—reaches the human cortex and increases cortico-spinal excitability by reducing GABA_A_ α5-mediated inhibition [6].

As no validated post-acute phase treatment exists to improve functional recovery after stroke events, no active comparator is available. In this context, a placebo comparator group is commonly employed and required by the guidelines to demonstrate efficacy in controlled clinical trials [10].

During the double-blind treatment period (D0 to D90), S 44819 or placebo is provided in the form of sachets (two in the morning and two in the evening) of identical appearance and taste for all treatment groups. Patients remain on the same IMP and dose throughout the treatment period. Depending on the patient’s condition, different methods of treatment administration are planned, e.g., with a glass of water or thickened water, if necessary through a nasogastric tube.

Criteria for discontinuing treatment are any adverse event according to the judgment of the investigator, any QTcF prolongation, any severe hepatic event, any suicide attempt, any symptomatic haemorrhagic stroke, or any new IS. Also, consent withdrawal or any event which could jeopardize the patient’s safety lead to treatment discontinuation. In such a situation, the withdrawal reason is reported and all examinations are expected to be performed.

Compliance with treatment is assessed at each study visit by deduction of the number of sachets dispensed and returned.

### Endpoints and measurements

The primary objective is to demonstrate the superiority of at least one of the two doses of S 44819 versus placebo on functional recovery after IS based on the modified Rankin Scale (mRS) [11] measured after 90 days of treatment. The mRS is administered at days 5, 30, 60, and 90 and at the follow-up visit (day 105).

The secondary objectives are to assess the efficacy of two doses of S 44819 versus placebo on stroke recovery using the National Institute of Health Stroke Scale (NIHSS) [12], on activities of daily living using Barthel Index (BI) total scores [13], and on cognitive performance tests (MoCA, TMT), as well as the safety and tolerability of S 44819.

The NIHSS is administered at selection visit, at baseline (day 0, inclusion), and at days 5, 30, 60, 90, and 105. The BI is administered at days 30, 60, 90, and 105.

Cognitive performance is assessed using the Montreal Cognitive Assessment scale (MoCA) [14] and Trail Making Test (TMT) [15]. The MoCA (total score) and TMT (A and B, times) results are obtained at days 30 and 90. In order to evaluate cognitive performances when the condition begins to stabilize, cognitive assessment is obtained at 1 month and 3 months to assess the course of cognitive impairment in each stroke patient.

A visual analog scale evaluates various sub-dimensions of the participant’s quality of life (appetite, sleep, day-time alertness, mood, anxiety, and pain).

Safety and tolerability assessment includes adverse event (AE) recording, vital signs, and physical examination at all visits; body weight at selection, days 30, 60, 90, and 105, BMI and ECG parameters at days 0, 5, 30, 60, 90, and 105, and different laboratory parameters at days 0, 30, 60, 90, and 105.

Suicidal ideation and suicidal behavior is assessed using the Columbia Suicide Severity Rating Scale (C-SSRS) [16] and is administered at each visit from days 5 to 105.

Measurements are also performed in case of premature withdrawal.

**Fig. 2:**
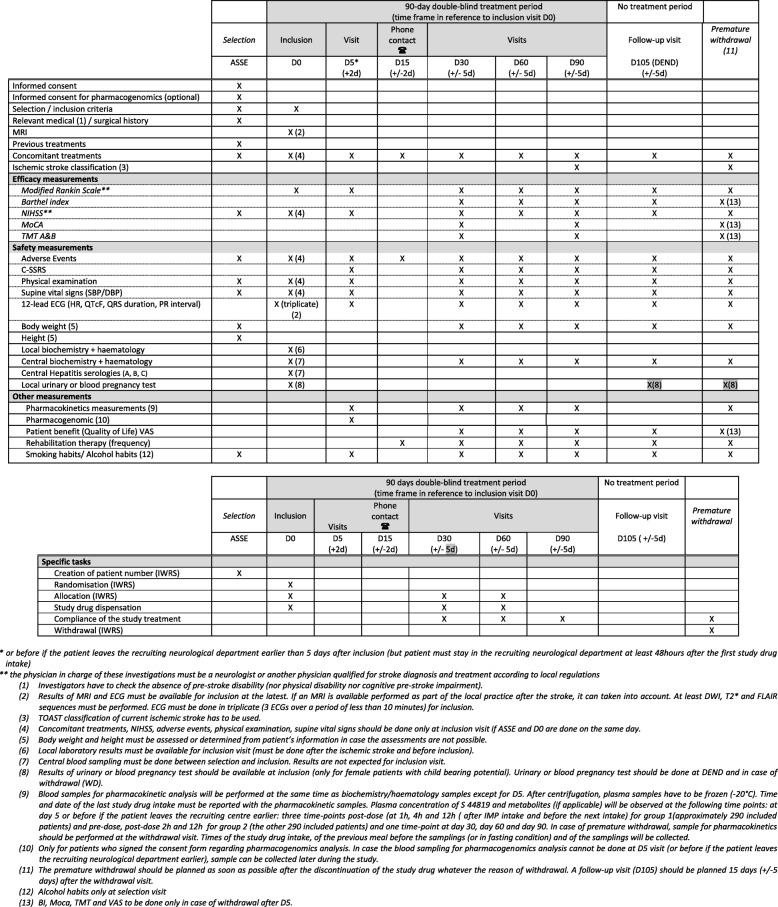
Standard Protocol Items: Recommendations for Interventional Trials (SPIRIT) figure

A SPIRIT figure is shown in Fig. 2 and a SPIRIT checklist is available in Additional file 1.

### Sample size estimates

The sample size (580 patients) was estimated based on the mRS score at day 90, using the last observation carried forward (LOCF) approach, to detect a treatment effect between at least one dose of S 44819 and placebo in the full analysis set (FAS) using Whitehead’s formula [17] for ordered categorical criteria. To maintain the experiment-wise type I error at 5%, the Bonferroni correction is to be applied. A drop-out rate of 5% until D5 was considered. This sample size should allow reaching the primary objective with a power of 85% if the true difference is 15 points in the cumulative proportion of patients with mRS 0–2 after 90 days of treatment and considering the following distribution of mRS assumed for placebo after 90 days of treatment:

**Table:** 

mRS	0	1	2	3	4	5–6
Placebo (%)	10	15	20	20	20	15

The placebo distribution was adapted from the NEST-1 trial [18] with similar inclusion criteria concerning severity (NIHSS score between 7 and 22). The results of NEST-1 show that such a placebo distribution is a reasonable estimation.

### Statistical analysis

#### Main analysis

The primary efficacy objective is assessed in the FAS (all patients of the RS=Randomised Set having taken at least one dose of treatment and having at least a value for the primary efficacy endpoint after D5) from the mRS score at day 90. An ordinal logistic regression assesses the odds of shifting from one category to the next on the mRS scale. Analysis includes the fixed, categorical effects of treatment (including the three treatment groups), country, and previous revascularisation therapy. Missing data are imputed with the LOCF approach. The step-down Dunnett procedure accounts for multiplicity of comparisons.

To assess the robustness of the primary analysis results with regard to the method of handling missing data, an ordinal logistic regression, including the fixed, categorical effects of treatment, country, and previous revascularisation therapy, is carried out in the FAS considering the multiple imputation method. The same analysis as the primary analysis is performed in the FAS, including in addition the continuous fixed, covariates of age, and baseline NIHSS score. Multiplicity is handled in the same way as in the primary analysis.

#### Secondary analyses

The same analyses as for the primary analysis are performed in patients of the per protocol set (all completed patient data of the FAS without relevant deviation). The difference between each S 44819 dose and placebo is studied in the FAS at day 90 on the dichotomized mRS scores (0–1 versus 2–6 and 0–2 versus 3–6) using a logistic regression including the fixed, categorical effect(s) of treatment, country, and previous revascularisation therapy. Missing data at day 90 are imputed with the LOCF approach. The step-down Dunnett procedure is used to account for multiplicity of comparisons.

Descriptive statistics by treatment group are provided for all analytical approaches of the primary efficacy endpoint in patients of the FAS. The mRS score is described at each visit in patients of the per protocol set.

##### Secondary endpoints

The secondary efficacy endpoints include 1) the value of the NIHSS score at baseline and at each post-baseline visit, 2) the value of the BI total score at each visit, 3) the value of the MoCA total score at each visit, and 4) the TMT times at each visit.

The difference between each S 44819 dose and placebo is studied in the FAS at day 90 for the NIHSS and BI scores using a Mann–Whitney test. Missing data at day 90 are imputed with the LOCF approach. Descriptive statistics by treatment group are provided for the NIHSS, BI, MoCA, and TMT endpoints in patients of the FAS.

Number of events and the number and percentage of patients reporting at least one adverse event are provided for serious and emergent adverse events. These events are described according to the seriousness, intensity, relationship, action taken regarding S 44819, the requirement of added therapy, time from onset, and outcome.

Vital signs, laboratory parameters, ECG parameters, and physical examination are described in terms of value at baseline, value at each post-baseline visit under treatment, last post-baseline values under treatment, and values at follow-up visit, as well as variations from baseline to each post-baseline visit under treatment and to the last post-baseline value under treatment.

For suicidal ideation score from the C-SSRS, the number and percentage of patients is assessed considering their maximum suicidal ideation score during treatment. Suicidal ideation score is also described at follow-up visit. For the other outcomes, the number and percentage of patients that reach each outcome during treatment (defined as a “yes” answer at any time during treatment) and at follow-up visit are described.

Patient quality of Life (QoL) is scored at each visit and descriptive statistics are reported for visual analogue scale scores for the D0–D90 period as well as at day 105.

Statistical analysis is performed using SAS® software, version 9.2 (Cary, NC, USA).

### Data Monitoring Committee

1A Data Monitoring Committee (DMC) is responsible for reviewing on a regular basis strictly confidential data related to the safety of patients participating in the study. Based on the review of the safety data, the DMC gives written recommendations to the sponsor about the continuation of the study per the protocol until the next DMC meeting, or about the continuation of the study with modification(s) that have no impact on the study design, or about the premature discontinuation of the study.

## Discussion

S 44819 exerts long lasting improvement of post-stroke recovery in rodent IS models when administered from day 3 after the ischemic insult [5] and has been shown to increase cortical excitability by decreasing GABA-mediated inhibition in healthy volunteers [6]. It is hypothesized that S 44819 may enhance post-stroke neuroplasticity in patients by counteracting enhanced cortical tonic inhibition.

Accumulating evidence suggests that there is a critical period of increased neuroplasticity during the early post-stroke recovery phase [3, 19] and it is crucial to initiate therapy during this time window. In this trial, S 44819 is administered to patients starting 3 to 8 days after stroke onset.

The study protocol considers all ethical principles of a placebo-controlled trial and the best standards of care. To increase the acceptability of the present study, the probability to receive placebo is fixed at 33%. Given that S 44819 is expected to improve recovery in IS patients regardless of their initial condition, a shift analysis is recommended [20]. Comparisons between S 44819 and placebo are assessed using ordinal logistic regression to assess the odds of shifting from one category to the next.

The mRS, NIHSS, and BI scales are analyzed as secondary efficacy scales; they are recommended by EMA guidelines to assess the efficacy of medicinal products for treating acute stroke [7].

S 44819 does not bind to benzodiazepine sites or to GABA_A_ receptors containing α1, α2, and α3 subunits [4] and presumably does not cause adverse effects triggered by these subunits; so far, no safety concern has arisen from the phase I and the on-going phase II study.

## Conclusion

This trial may provide the first proof of concept for an innovative therapeutic approach based on the enhancement of functional recovery after IS.

### Trial status

Protocol version 7, 23/07/2018. The study started in Dec 2016 and was completed in March 2019.

## Supplementary information

**Additional file 1.** SPIRIT 2013 checklist: Recommended items to address in a clinical trial protocol and related documents.

## Data Availability

Anonymized patient-level, study-level clinical trial data (including clinical study report) and study protocol underlying the results reported in this article will be shared in agreement with the Servier Data Sharing Policy available at https://clinicaltrials.servier.com/data-request-portal/. Access to data will be granted to researchers identified in the research proposal directed to https://clinicaltrials.servier.com/data-request-portal/, to achieve the aims described in this proposal, and provided it is approved by a dedicated committee and a signed data sharing agreement by the requestor is provided.

## Abbreviations

AE: Adverse events
BI: Barthel Index
C-SSRS: Columbia Suicide Severity Rating Scale
FAS: Full analysis set
IS: Ischemic stroke
IWRS: Interactive Web Response System
LOCF: Last observation carried forward
MoCA: Cognitive Assessment scale
mRS: Modified Rankin Scale
NIHSS: National Institute of Health Stroke Scale
QoL: Quality of Life
QTcF: QT interval corrected using Fridericia formula for heart rate
TMS: Transcranial magnetic simulation
TMT: Trail Making Test
